# Proteomic Interrogation of Human Chromatin

**DOI:** 10.1371/journal.pone.0024747

**Published:** 2011-09-14

**Authors:** Mariana P. Torrente, Barry M. Zee, Nicolas L. Young, Richard C. Baliban, Gary LeRoy, Christodoulos A. Floudas, Sandra B. Hake, Benjamin A. Garcia

**Affiliations:** 1 Department of Chemistry, Princeton University, Princeton, New Jersey, United States of America; 2 Department of Molecular Biology, Princeton University, Princeton, New Jersey, United States of America; 3 Department of Chemical Engineering, Princeton University, Princeton, New Jersey, United States of America; 4 Munich Center for Integrated Protein Science and Adolf-Butenandt-Institute, Department of Molecular Biology, Ludwig Maximilians University of Munich, Munich, Germany; University of Virginia, United States of America

## Abstract

Chromatin proteins provide a scaffold for DNA packaging and a basis for epigenetic regulation and genomic maintenance. Despite understanding its functional roles, mapping the chromatin proteome (i.e. the “Chromatome”) is still a continuing process. Here, we assess the biological specificity and proteomic extent of three distinct chromatin preparations by identifying proteins in selected chromatin-enriched fractions using mass spectrometry-based proteomics. These experiments allowed us to produce a chromatin catalog, including several proteins ranging from highly abundant histone proteins to less abundant members of different chromatin machinery complexes. Using a Normalized Spectral Abundance Factor approach, we quantified relative abundances of the proteins across the chromatin enriched fractions giving a glimpse into their chromosomal abundance. The large-scale data sets also allowed for the discovery of a variety of novel post-translational modifications on the identified chromatin proteins. With these comparisons, we find one of the probed methods to be qualitatively superior in specificity for chromatin proteins, but inferior in proteomic extent, evidencing a compromise that must be made between biological specificity and broadness of characterization. Additionally, we attempt to identify proteins in eu- and heterochromatin, verifying the enrichments by characterizing the post-translational modifications detected on histone proteins from these chromatin regions. In summary, our results provide insights into the value of different methods to extract chromatin-associated proteins and provide starting points to study the factors that may be involved in directing gene expression and other chromatin-related processes.

## Introduction

Chromatin plays a key role in nearly all eukaryotic DNA templated processes such as mitosis, DNA repair, and transcription. Disruption of chromatin structure is intimately associated with various human diseases, such as cancer and several congenital syndromes including α-thalassemia/mental retardation and Rubinstein-Taybi syndromes [1], [2]. The molecular basis for chromatin function can be understood at level of the nucleosome, comprised of approximately 146 base pairs of DNA coiling around an histone octamer conformed by one histone H3–H4 tetramer and two histone H2A–H2B dimers [3]. Chromatin domains are formed and maintained by the interaction and post-translational modification (PTM) of chromatin proteins, which can epigenetically alter gene expression [4], [5], [6]. Within these domains, transcriptionally active regions constitute euchromatin, while transcriptionally inert regions constitute heterochromatin [7], [8]. Euchromatin is less condensed, and believed to be more accessible to transcription factors, whereas heterochromatin is more condensed and less accessible to the transcriptional machinery [7]. This epigenetic and structural regulation, along with the genomic information, has been termed the “Chromatome” [9]. Improved system-wide knowledge of the components of chromatin could provide a holistic insight into its higher-order structure and function.

Fully characterizing the chromatome is nontrivial as many chromatin proteins are expressed transiently, at low levels, or are difficult to extract from the nucleus [10]. Furthermore, no purification method has arisen as the “gold standard” for chromatin extraction. Proteomic techniques have partly circumvented these difficulties and considerably accelerated studies on the chromatin proteomes from various species including *Oryza sativa*, *Saccharomyces cerevisiae*, *Xenopus laevis*, and *Caenorhabitis elegans* [11], [12], [13], [14]. Several mass spectrometry (MS)-based proteomic studies have also made notable progress in characterizing human chromatin from mitotic chromosomes [15], [16]. In B lymphocytes, over 280 chromatin proteins were recently identified, however, with only 64 known to be nuclear, clearly illustrating the technical issues associated with purifying chromatin fractions [10]. While a vast number of chromatin proteins has been detected, the total number of human chromatin proteins, including variants and isoforms, is likely to be much larger with over 2,000 hypothetical human genes encoding for transcriptional activators alone [17], [18].

Another layer of chromatome complexity lies in the post-translational modification (PTM) of chromatin proteins. Several chromatin proteins are known to be highly modified, such as Heterochromatin Protein 1 (HP1) and High Mobility Group (HMG) proteins, where these PTMs may control protein function and regulate chromatin structure [19], [20], [21]. Notable among this broad class of proteins, histone proteins exhibit extensive PTM patterns including methylation and acetylation at specific residues [3], [22]. Interestingly, histone PTMs are linked to various cellular events including apoptosis, cellular differentiation, and the cell cycle [3], [23], [24]. Specific histone PTMs have been reported to associate with eu- and heterochromatin, and coexisting PTMs form and maintain those regions [25], [26], [27]. The diversity and specificity associated with histone PTMs has led to the ‘Histone Code’ hypothesis, which proposes that PTMs act as binding sites for other chromatin proteins that “interpret” these modifications to regulate DNA-templated processes [22], [28], [29]. Quantifying the full collection of histone PTMs involved in euchromatin and heterochromatin maintenance therefore may illuminate downstream and upstream mechanisms governing these genomic regions.

Here we present a large-scale proteomic mass spectrometry-based comparison of three selected chromatin extraction methods. Proteins enriched in each preparation were analyzed via a Bottom Up mass spectrometry based proteomics approach including separation by one-dimensional gel electrophoresis (1D-SDS-PAGE), in-gel tryptic digestion, and nanoflow LC-MS/MS performed in a high-resolution Orbitrap mass spectrometer. Our results indicate that, depending on the downstream application, a decision between biological specificity and broadness of characterization must be made in selecting a chromatin purification method. By way of this qualitative comparison, we also achieve an extensive proteomic catalog of human chromatin. This platform identified over 1,900 unique proteins from these fractions, the majority of which are annotated as nuclear proteins. We analyzed our datasets using a Normalized Spectral Abundance Factor (NSAF) approach to obtain a relative protein abundance profile and also detected numerous PTMs in our datasets, including acetylation, mono-, di- and trimethylation of lysines and arginine methylation [30]. Moreover, we attempted to carry out proteomic investigations into euchromatin- and heterochromatin-enriched fractions and identified proteins seemingly enriched in either fraction. To corroborate the enrichment for these genomic regions, we also characterized histone PTMs in the euchromatin or heterochromatin enriched samples using a stable isotope labeling quantitative MS method. We hope our findings will act as a foundation for additional studies involving the higher-level structure of chromatin and its roles in basal or aberrant gene functions during dynamic or epigenetic processes.

## Materials and Methods

All chemicals and reagents were purchased from Sigma Aldrich (St. Louis, MO) unless otherwise noted.

### Cell Culture

HeLa S3 cells were grown and harvested as previously described [31].

### Total Chromatin Extraction

Chromatin was isolated as described with the following modifications [10], [32]. Cells were resuspended in Buffer A (10 mM HEPES pH = 7.9, 10 mM KCl, 1.5 mM MgCl_2_, 0.34 M sucrose, 10% glycerol, inhibitor cocktail: 1 mM DTT, 0.5 mM 4-(2-aminoethyl) benzenesulfonyl fluoride hydrochloride, 5 mM microcystin and 10 mM sodium butyrate). Triton X-100 was added to a final concentration of 0.1% and the suspension was incubated for 8 minutes on ice. The nuclear pellet was obtained by centrifugation (1,300× g for 5 minutes at 4°C), washed with Buffer A and then resuspended in Buffer B (3 mM EDTA, 0.2 mM EGTA, inhibitor cocktail) for 30 minutes on ice. The insoluble chromatin pellet was isolated by centrifugation (1,700× g for 5 minutes at 4°C) and then resuspended in 15 mM Tris, pH = 7.5, 0.5% SDS.

### Salt Extraction of Chromatin

Cells were resuspended in hypotonic lysis buffer (10 mM HEPES/KOH pH = 7.9, 1.5 mM MgCl_2_, 10 mM KCl and inhibitor cocktail) and incubated on ice for 30 minutes. Nuclei were isolated by centrifugation (4,000 rpm for 10 minutes at 4°C), and the supernatant was discarded. The nuclear pellet was resuspended in high salt buffer (20 mM HEPES/KOH pH = 7.9, 25% glycerol, 420 mM KCl, 1.5 mM MgCl_2_, 0.2 mM EDTA, and inhibitor cocktail) and sonicated for 30 seconds (3×10 s) on ice. The suspension was rotated at 4°C for 2 hours and then centrifuged (4,000 rpm for 20 minutes at 4°C). The salt concentration of the supernatant was lowered through a 5-fold dilution with minimal-salt buffer (20 mM HEPES/KOH pH = 7.9, 25% glycerol, 1.5 mM MgCl_2_, 0.2 mM EDTA and protease inhibitors). The pellet was resuspended and dialyzed overnight at 4°C against minimal-salt buffer in a Slide-A-Lyzer® Dialysis Cassette (Pierce Biotechnology, Rockford, IL).

### Chromatin Extraction through Micrococcal Nuclease Digestion

Nuclei were isolated as previously described [33]. Briefly, cells were lysed in Nuclear Isolation Buffer (NIB, 15 mM Tris pH = 7.5, 15 mM NaCl, 60 mM KCl, 5 mM MgCl_2_, 1 mM CaCl_2_, 250 mM sucrose and inhibitor cocktail) supplemented with 0.3% NP-40 (Calbiochem, EMD Biosciences, La Jolla, CA). The resulting nuclei pellet was separated by centrifugation (600× g for 5 minutes at 4°C) and washed twice with NIB. Micrococcal nuclease (MNase) digestion was performed as described with the following modifications [34]. Nuclei were resuspended in NIB to a concentration of approximately 10^7^ nuclei/mL and then preincubated at 37°C for 10 minutes. MNase was added to a final concentration of 5 units/mL. The digestion proceeded at 37°C for 20 minutes with occasional mixing, and quenched with 50 mM EDTA. Finally, the sample was centrifuged at full speed in a tabletop microcentrifuge at 4°C for 10 minutes to obtain a supernatant and a pellet.

### Euchromatin and Heterochromatin Extraction through Partial MNase Digestion

Chromatin was isolated as described before with minor changes [35]. Micrococcal nuclease was added to a final concentration of 1.2 units/mL, and the reaction was quenched by 1 mM EGTA on ice for 10 minutes. The sample was centrifuged at 1,000× g for 5 minutes at 4°C to generate the first supernatant (S_1_). The pellet was resuspended in 2 mM EDTA at pH = 7.2 in the same volume as S_1_ and incubated on ice for 10 minutes. The sample was then centrifuged at 12,000× g at 4°C to yield a second supernatant (S_2_) and a pellet. Resulting fractions were loaded onto a 12% SDS polyacrylamide gel (SDS-PAGE).

### In-Gel Digestion of Chromatin Proteins

Approximately 100 µg of extract from each fraction was resolved on a 12% SDS-PAGE gel. Each lane was cut into 10 slices containing approximately the same amount of protein by visual inspection. In-gel digestion was performed according to the protocol described previously with minor modifications [36]. Each sample was desalted on a C_18_ StageTip prior to MS [37].

### Extraction of DNA

DNA was isolated from chromatin samples by chloroform/phenol extraction as described previously [38]. The DNA was ethanol-precipitated and then resuspended in water and loaded onto a 2% agarose gel.

### Histone Extraction and Separation

Histones were extracted from S_1_ and S_2_ fractions with 0.4N H_2_SO_4_ and precipitated with trichloroacetic acid (TCA), followed by washes with acetone+0.1% HCl and then acetone [33]. Bulk histones were redissolved in water and fractionated on a C_18_ column (4.6 mm i.d.×250 mm, Vydac) using an Beckman Coulter System Gold HPLC (Fullerton, CA) with a gradient of 30–60% B in 100 min, followed by 60–100%B in 20 min (*A* = 5% acetonitrile (MeCN) in 0.2% trifluoracetic acid (TFA), *B* = 90% MeCN in 0.188% TFA) [39]. Fractions were dried to completion in a vacuum centrifuge and checked for purity by 15% SDS-PAGE.

### Histone Preparation for Bottom Up MS

HPLC purified histone variants (<5 µg) were derivatized with propionyl anhydride as described before [40]. For quantification studies, either the euchromatic or heterochromatic histones were labeled using *d_10_*-propionic anhydride both before and after trypsin digestion to introduce a +5Da mass shift (Cambridge Isotope Laboratories, Andover, MA) [41]. For comparative MS analysis, protein concentrations of each sample were determined using Bradford reagent to ensure equal mixing.

### NanoLC-MS/MS

Peptides were eluted from C_18_ Stage Tips using 75% MeCN, 5% acetic acid; the acetonitrile was subsequently evaporated through vacuum centrifugation [37]. All MS experiments were performed in the following manner. Peptides were loaded by an Eksigent AS-2 autosampler (Eksigent Technologies, Dublin, CA) onto a fused silica microcapillary (75 µm) column packed in-house with 5 µm C_18_ YMC ODS-A resin constructed with an integrated ESI tip. Loaded peptides were HPLC separated with an Agilent 1200 series binary pump across a 150-min linear gradient ranging from 2% to 35% buffer B (Buffer A = 0.1 M acetic acid, Buffer B = 70% MeCN in 0.1 M acetic acid) with a flow of 100–200 nL/min. For histones, a 110-min gradient was used. The HPLC was coupled to an LTQ-Orbitrap mass spectrometer (ThermoFisher Scientific, Waltham, MA). Full MS spectra (*m/z* 300–1650) were acquired in the Orbitrap with a resolution of 30,000 at *m/z* 400 after accumulation of 500,000 ions. The seven most intense ions were sequenced by collision-induced dissociation (normalized collision energy 35%) in the LTQ after accumulation of 10,000 ions concurrently to full scan acquisition in the Orbitrap. Maximal filling time was 500 ms for the full scans. Precursor ion charge state screening was enabled and all unassigned charge states as well as singly charged species were rejected. The dynamic exclusion list was restricted to a maximum of 500 entries with a maximum retention period of 120 seconds and a relative mass window of <1 Da.

### Data Analysis

Mass spectra were searched using the SEQUEST algorithm within the Bioworks Browser (Version 3.3.1 SP1, Thermo Fisher Scientific) against the National Center for Biotechnology Information (NCBI) human protein database. Three missed cleavage sites were allowed. Peptide tolerance was set to 0.1 Da and fragment ion tolerance was set to 0.5 Da. Carboxyamidomethylation on cysteine (+57.021) was set as a fixed modification, while oxidation of methionine (+15.999) was set as a variable modification. For PTM searches, acetylation (+42.010 Da), mono- (+14.016 Da), di- (+28.031 Da) and trimethylation (+42.046 Da) of lysine residues, mono (+14.016 Da) and dimethylation (+28.031 Da) of arginine residues, and N-terminal acetylation (+42.010 Da) were selected as variable modifications. Resulting peptides were filtered using criteria as previously described, such as Xcorr values of 2, 2.5, and 3 for charge states of 2, 3, and 4 respectively [42]. Protein matches with a probability higher than 5×10^−3^ were not considered. The false positive rate was estimated to be the 1% level by searching a reverse database as previously stated [43].

To quantify relative protein abundance, we calculated the Normalized Spectral Abundance Factor (NSAF) for each protein. We developed a script in Matlab R2007b (Version 7.5.0.342, August 2007, The MathWorks, Inc.) that counts the number of tandem mass spectra for a given protein and obtains the information to calculate spectral abundance as previously published. Proteins identified from only a single MS/MS spectrum were discarded. For euchromatin and heterochromatin analysis, only proteins found multiple times in biological and technical replicates were included in the results. Functional annotation for more than 80% of the identified proteins was carried out with the online tool DAVID Bioinformatic Resources 2008 (http://david.abcc.ncifcrf.gov/) [44], [45], [46]. Default settings in DAVID's functional annotation tool were used to search each dataset. For histone PTM determination, spectra were manually analyzed. Fold change was calculated by taking the abundance of a given modification in euchromatin and dividing it over the abundance of the same modification in heterochromatin. A fold change higher than 1 would indicate enrichment for the modification in euchromatin, while a fold change lower than 1 would indicate enrichment for the modification in heterochromatin. Heat maps depicting the ratio of histone PTMs in euchromatin over heterochromatin were created by using Java Treeview and Matlab [47]. Fisher's exact test was performed to determine the statistical significance associated with the enrichment of euchromatic and heterochromatic histone PTMs collectively in the S_1_ and S_2_ fractions, respectively.

## Results and Discussion

### Large-scale proteomic analyses of chromatin enriched protein fractions

**Figure 1:**
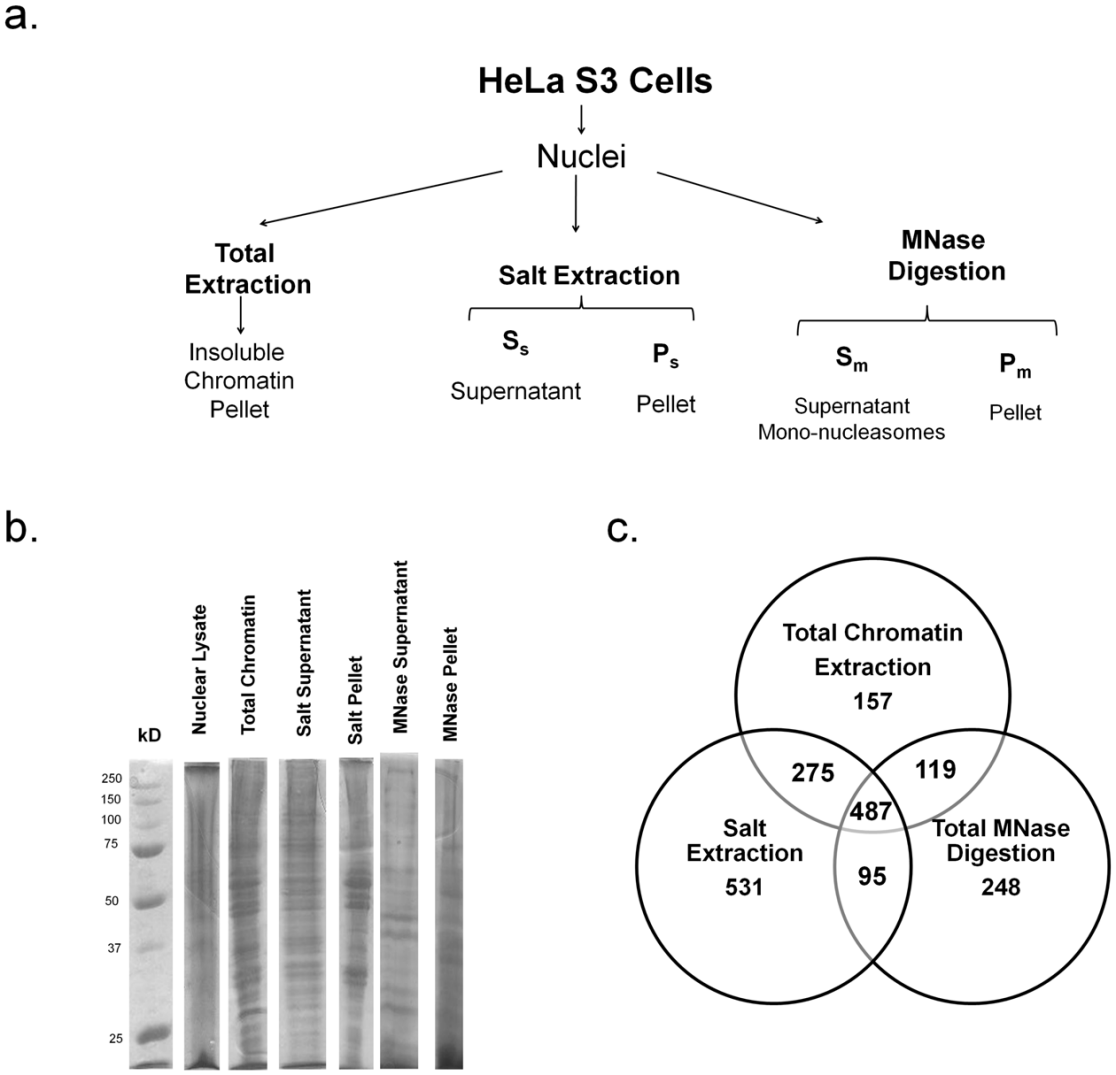
Comparison between chromatin purification methods. (a.) Chromatin was purified from HeLa cells through three different preparations. (b.) SDS-PAGE analysis of a crude nuclear lysate and the chromatin preparations. Approximately 10 ug of protein were loaded in each lane. Supernatant and pellet fractions are shown separately for the salt extraction and the MNase digestion protocols. (c.) Venn Diagram indicating the number of proteins detected in one, two or all three chromatin preparations. Where applicable, preparation fractions (supernatant and pellet) were combined.

To compare some preparations for chromatin-associated proteins, we extracted chromatin from HeLa S3 cells using a total chromatin extraction, a salt extraction and a total micrococcal (MNase) digestion as shown in **Figure 1a**, with the expectation that we would possibly detect slightly different subsets of chromatin proteins between the three methods. As shown by 1D-SDS-PAGE, each preparation enriched for different chromatin proteins (**Figure 1b**). We then analyzed the resulting chromatin samples (two technical replicates) from all three methods using mass spectrometry reporting only the proteins detected in both technical replicates. Through these analyses we found a total of over 77,000 peptides matching to 1,038 non-redundant proteins in the total chromatin extraction, 1,388 proteins in the salt extraction method (supernatant and pellet combined) and 949 proteins in the total MNase digestion method (supernatant and pellet combined, **Data S1**). All in all, these hits correspond to a total of 1,912 unique proteins in these chromatin enriched fractions including 193 previously uncharacterized (“hypothetical” or “predicted”) proteins (**Figure 1c** **, and Data S1**). In this report, a unique protein is a protein hit assigned an NCBI annotation number; thus, protein isoforms and protein complexes subunits are considered distinct. Approximately 25% of the proteins (487 hits) were purified across all three methods, while roughly half of the protein total seemed exclusive to a single preparation (**Figure 1c**). 261 of the proteins found in our screen were also listed in the chromatin database ChromDB which contains a total of 408 human chromatin proteins mapping to 466 protein accession numbers [48].

**Figure 2:**
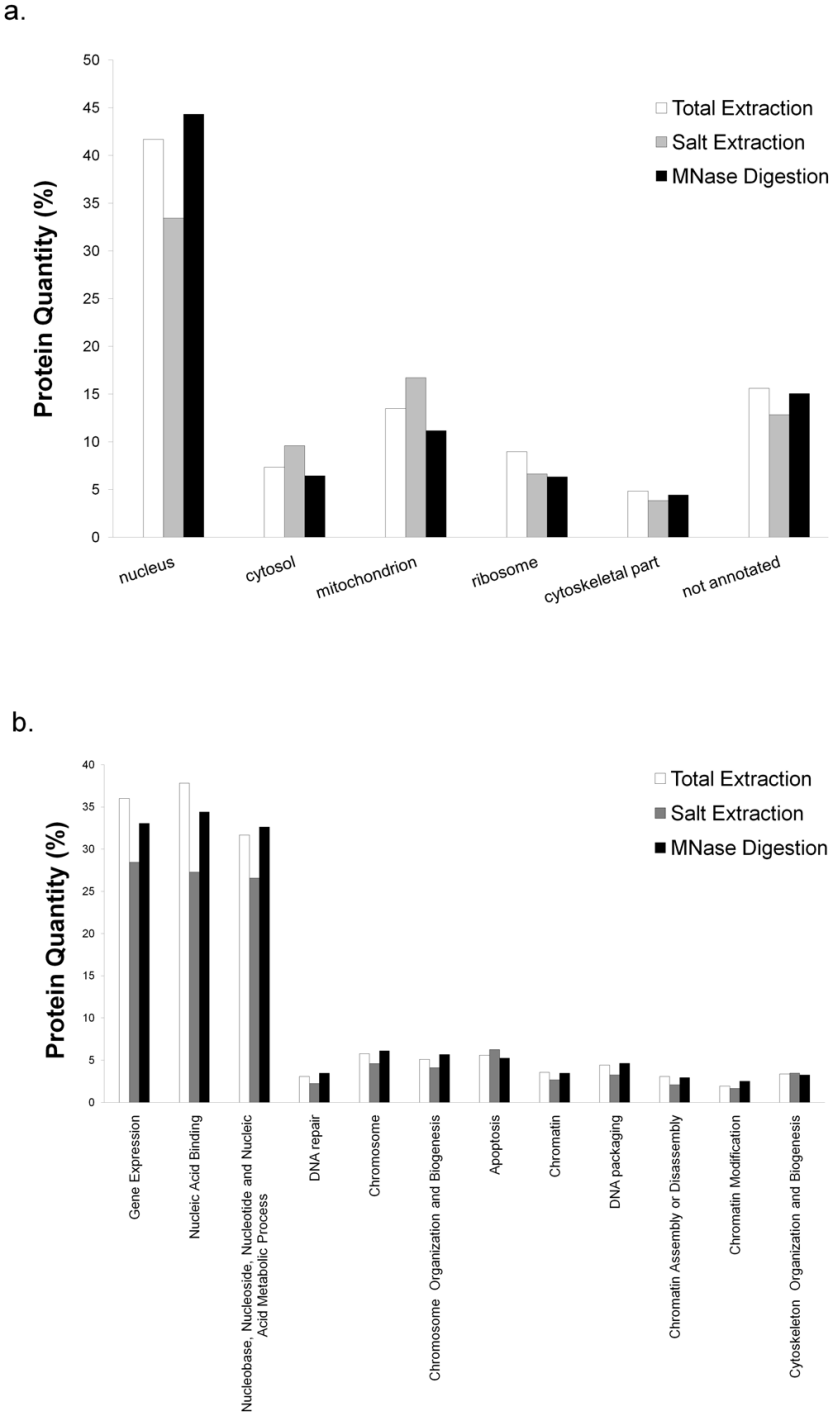
Classification of proteins identified in chromatin preparations. This was accomplished according to (a.) their cellular location and (b.) their functional categorization. The white column corresponds to the total extraction of chromatin, while gray and black columns correspond to the salt extraction and total digestion of chromatin respectively. Preparation fractions (supernatant and pellet) were combined where applicable. Protein quantity refers to the percentage of proteins in each category with respect to the total number proteins found in each particular preparation.

Over 30% of the proteins detected in all three data sets correspond to annotated nuclear proteins, while less than 10% are cytosolic. Fewer than 20%, 10%, and 5% of the non-nuclear proteins were annotated as mitochondrial, ribosomal and cytoskeletal respectively (**Figure 2a**). Almost 45% of the proteins identified in the total MNase digestion fraction are classified as nuclear, while lower percentages were observed in the other two preparations (**Figure 2a**). Uniquely, the MNase digestion detected known chromatin components of lower abundance such as Aurora Kinase B, SUV39H1 and other chromatin modifying proteins (**Table 1** **and Data S2**). The total extraction method identified fewer proteins than the salt extraction method, but was more specific for chromatin proteins (**Figure 2a**). Approximately 15% of the proteins found in each preparation could not be annotated, which is likely attributed to proteins with unknown or multiple cellular localizations (**Figure 2a**). We also examined the functional annotation of the identified proteins (**Figure 2b**). As expected, most proteins were involved in DNA processes, such as gene expression and nucleic acid biology. We also found proteins involved in biological events, such as DNA repair and apoptosis (**Figure 2b**). In agreement with our cellular localization data, the total chromatin extraction and the MNase digestion preparation have the highest proportion of proteins involved in chromatin processes (**Figure 2b**), with the MNase procedure demonstrating higher specificity for chromatin proteins. We believe that this is due to the utilization of a biological property of the target proteins, i.e. its association with DNA, rather than a nonspecific physical property, such as solubility. However, it is important to note that no protein annotation approach should be taken without caution. Namely, an issue with categorizing proteins is that a large number of proteins have multiple assigned cellular localization or function, and hence results are mixed or at best diluted.

**Table 1:** Relative quantification for selected chromatin-enriched proteins.

	Normalized Spectral Abundance Factor				
Protein	Total Extraction	Salt Extraction Sup.	Salt Extraction Pellet	MNase Dig. Sup.	MNase Dig. Pellet
H1	1.5E-02	9.2E-03	2.6E-02	5.6E-03	2.1E-02
H2A	3.5E-02	1.1E-02	2.0E-02	2.0E-02	1.8E-02
H3	2.3E-02	2.9E-03	1.0E-02	4.2E-03	1.1E-02
H4	3.6E-02	1.2E-02	2.9E-02	2.2E-02	3.4E-02
H2B	2.8E-02	6.3E-03	1.8E-02	1.4E-02	2.7E-02
Core histone macroH2A2.2	1.4E-03	n/d^1^	n/d	7.7E-04	1.7E-04
HP1 alpha	9.2E-04	4.2E-04	2.0E-03	1.7E-03	8.5E-04
HP1 beta	4.3E-04	1.1E-03	2.2E-03	9.0E-03	1.6E-03
HP1 gamma	8.2E-04	2.9E-04	1.4E-03	3.4E-03	1.6E-03
Histone deacetylase 1	1.8E-04	n/d	2.4E-04	n/d	6.1E-04
Histone deacetylase 2	1.4E-04	1.8E-04	n/d	4.9E-04	3.3E-04
chromatin-specific transcription elongation factor large subunit	2.9E-04	3.5E-04	1.8E-04	5.9E-04	1.6E-03
general transcription factor II, isoform 3	1.9E-04	3.8E-04	n/d	n/d	n/d
SWI/SNF-related matrix-associated actin-dependent regulator of chromatin	8.4E-05	n/d	n/d	7.24E-04	1.6E-03
chromatin assembly factor 1 subunit B	7.9E-05	n/d	n/d	2.6E-04	n/d
upstream binding transcription factor, RNA polymerase I isoform b	4.8E-05	2.6E-04	n/d	n/d	4.4E-04
transcription factor MAFF	1.1E-04	n/d	n/d	n/d	n/d
transcription factor ELYS	7.8E-06	n/d	n/d	n/d	1.0E-04
general transcription factor IIF, polypeptide 2 (30kD subunit)	n/d	5.3E-04	n/d	n/d	n/d
DNA (cytosine-5-)-methyltransferase 1	6.5E-05	4.9E-05	n/d	8.8E-05	n/d
O-6-methylguanine-DNA methyltransferase	2.1E-04	5.1E-04	n/d	n/d	n/d
chromatin accessibility complex 1	n/d	n/d	2.9E-04	n/d	n/d
suppressor of variegation 3–9 homolog1	n/d	n/d	n/d	n/d	3.9E-04
centromere protein F (350/400kD)	n/d	n/d	n/d	4.6E-05	n/d
chromatin modifying protein 4B	n/d	n/d	n/d	n/d	8.7E-04
chromatin modifying protein 4A	n/d	n/d	n/d	n/d	2.4E-04
Aurora kinase B	n/d	n/d	n/d	6.9E-04	4.7E-04
DNA directed RNA polymerase II polypeptide E	2.9E-04	5.0E-04	5.4E-04	1.1E-03	6.1E-04

1 Not detected.

To semi-quantitatively measure the relative abundance of the identified proteins, we used a Normalized Spectral Abundance Factor (NSAF) approach [30]. Not surprisingly, we found the most abundant proteins in the total chromatin extraction included the four core histones (H2A, H2B, H3 and H4) and H1 (**Table 1**, **Data S2** and **Figure S1**). We detect the histone proteins among the most prevalent hits across all three methods. These findings make much sense as core and linker histone proteins are estimated to constitute approximately 70% of chromatin [16]. Other predominant proteins present in our dataset include heterochromatin protein 1 (HP1), high mobility group (HMG) proteins, and RNA polymerase II, all well-characterized nuclear proteins (**Data S2**). While RNA polymerase II is involved in DNA transcription [49], HP1 and HMG proteins are involved in regulating chromatin structure and accessibility: HP1 binds histone H3 methylated at Lys 9 and promotes gene silencing and heterochromatin formation [8] and HMG proteins associate with nucleosomes to modulate specific gene expression [50].

We also identified proteins involved in DNA methylation such as Cytosine-5-methyl-transferases, in DNA damage recognition such as DNA damage binding protein 1, in binding specific histone PTMs such as bromo- and chromo-domain containing proteins, and in modifying histones, such as the enzymes poly-ADP ribose polymerase and HDACs (**Data S2**). As it is common in this type of large-scale survey analysis, we also detect proteins that are improbable chromatin proteins and more likely to be contaminants. The presence of non-nuclear proteins is inevitable due to their greater abundance relative to chromatin proteins. Interestingly, among the most abundant proteins extracted were actin and vimentin, proteins which have been traditionally considered to be cytoskeletal. However, recent evidence points towards their potential functional involvement in the nucleus. Actin has been demonstrated to be involved in chromatin remodeling and gene repositioning in the nucleus [51], [52]. Vimentin has been shown to bind DNA and has also been suggested to be involved in gene regulation events [53]. Therefore, with this data we cannot completely rule out normally classified cytoplasmic proteins as having a dual role in nuclear events, though further experimental validation is needed to definitely assign a nuclear function.

### Post-translational modifications on chromatin enriched proteins

**Figure 3:**
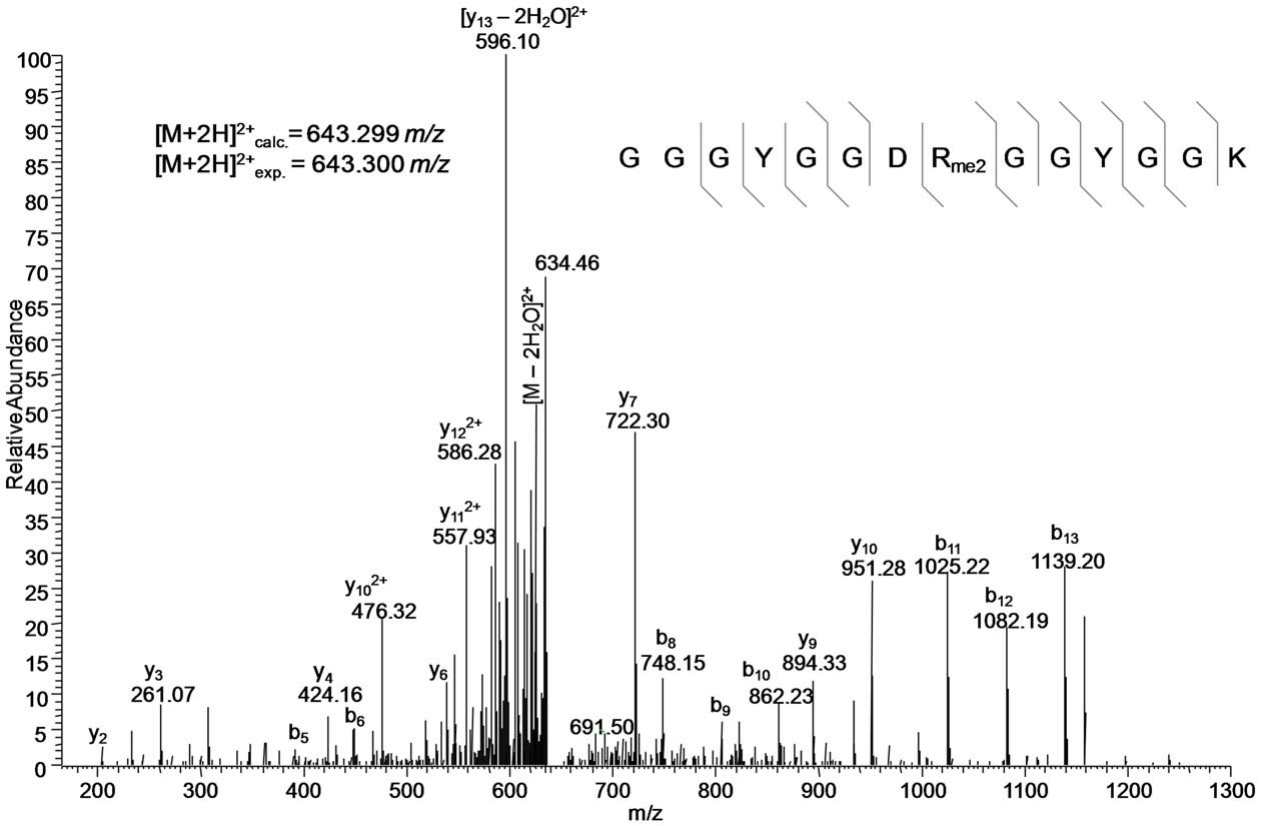
Post-translational modification of a chromatin-associated protein. MS/MS spectrum of the [M+2H]^2+^ precursor ion of TAF 2N isoform 2 (473–487 residue peptide) dimethylated on R480. Broken bonds above and below sequence denote b and y ions, respectively, that were annotated from the spectrum.

To characterize post-translational modifications (PTMs) on chromatin associated proteins, we examined our total chromatin datasets using the SEQUEST algorithm and modified peptides are listed in **Data S3**. All chromatin preparations preserved PTMs to a comparable degree. We confirmed several well documented cases of lysine and arginine methylation on histones H2A, H2B, H3, H4 and H1 and lysine methylation on HP1 [20], as well as potentially discovering novel modifications. For example, we detected dimethylation on R480 of TATA-binding protein-associated factor (TAF) 2N isoform 2, and to the best of our knowledge, this represents a novel assignment (**Figure 3**). Other sites of modification on the same protein include dimethylation of R203, R525, R532, R567, and monomethylation on R559 (**Data S3**). A previous proteomics study also identified R203me2 in the same cell line used in our study, corroborating our findings [54]. Overall, we found 45 proteins containing arginine mono- or dimethylation, 85 proteins containing lysine formylation, mono-, di-, or trimethylation, and 110 proteins containing lysine or N-terminal acetylation. Within the aforementioned classes, we found over 90, 155, and 130 unique post-translationally modified peptides, respectively. Since the number of modified peptides exceeds the number of modified proteins, several proteins contain multiple modification types and/or modified residues, such as the previously mentioned TAF2N. We excluded all C-terminally monomethylated peptides from our results, which may originate from an acid-catalyzed reaction between the C-terminus of the tryptic peptide and methanol in the sample preparation [55]. Although we chose to err on the rigorous side, this exclusion also means that our list probably underestimates the actual *in vivo* list of monomethylated proteins. Other higher degrees of methylation, as well as formylation and acetylation on C-terminal residues were included, as there is no facile explanation for their generation by protocol-induced chemical means, and they have been previously shown to be able to be cleaved by trypsin under prolonged digestion [56], [57]. Given that over 10% of the proteins identified between the three methods contain at least one PTM, it is likely that the PTMs serve specific chromatin biology function, possibly to regulate protein stability, conformation, localization, and interaction with DNA or other proteins [21], [51]. The last possibility is especially intriguing for TAF 2N isoform 2, where arginine methylation may influence the binding of TATA binding protein, TFIID complex or RNA polymerase II during initiation of transcription [58]. Further experiments, such as site-directed mutagenesis combined with functional readout, are needed to determine the significance of these modifications.

### Proteomics of euchromatin and heterochromatin enriched fractions

**Figure 4:**
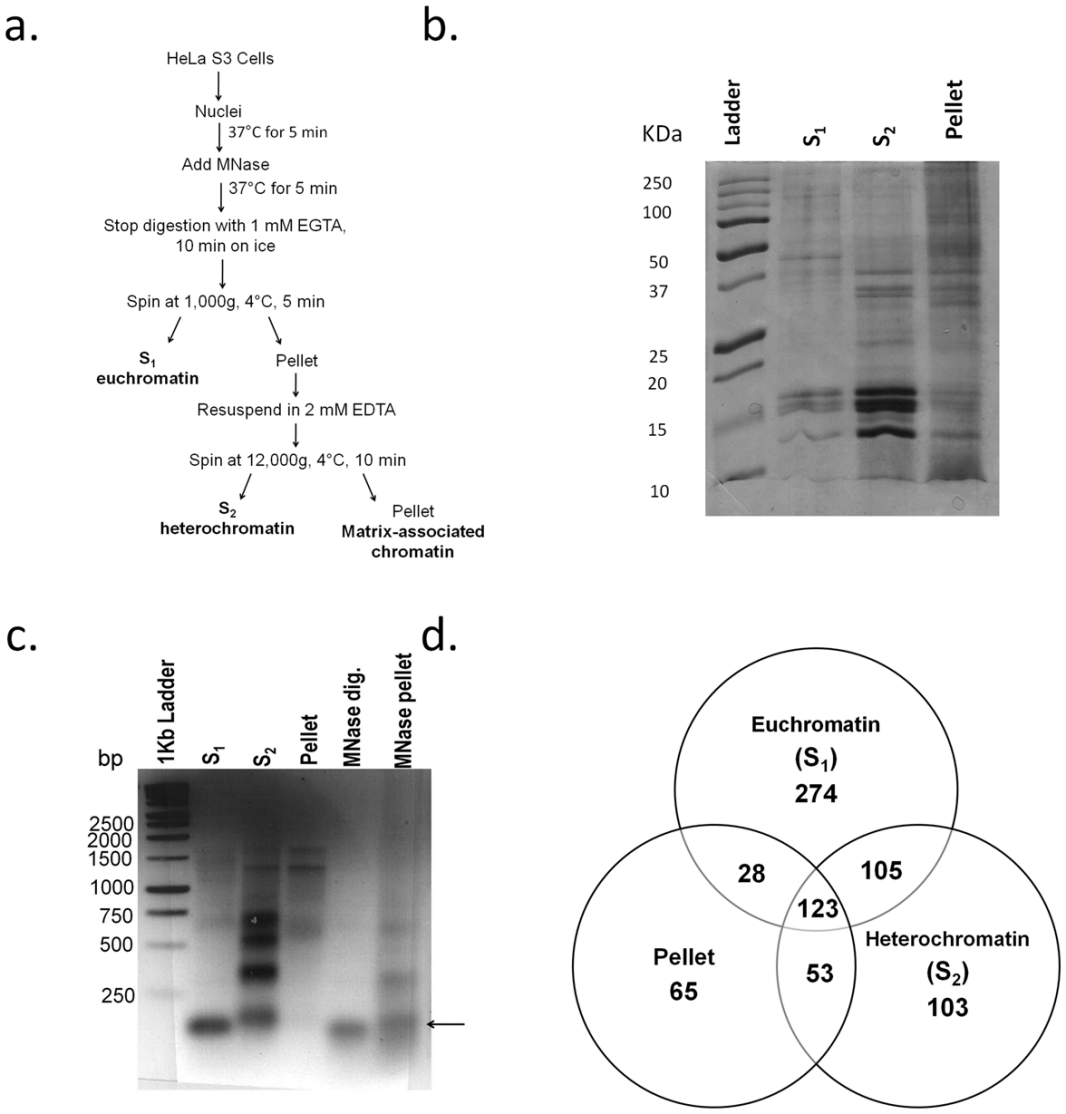
Crude separation of euchromatin and heterochromatin. (a.) Purification diagram, (b.) One-dimensional SDS-PAGE analysis of euchromatin (S_1_), heterochromatin (S_2_) and pellet fractions. Proteins in these fractions were separated by one-dimensional 15% SDS-PAGE gel and stained with Coomassie Brilliant Blue. Loading is approximately 10 ug of protein per lane, (c.) DNA gel (2% agarose) of S_1_, S_2_, and pellet fractions. S_1_ is composed mostly of mononucleosomes running at approximately 150 bp (black arrow). S_2_ and pellet fractions contain longer pieces of DNA corresponding to more compact chromatin. Total MNase digestion is shown for comparison, (d.) Venn Diagram indicating the number of proteins detected in one, two or all three fractions.

To further assess the potential of the MNase purification method and continue to define the profile of chromatin proteins, we attempted to identify proteins enriched in crude euchromatin and heterochromatin subfractions. MNase has long been used to study the structural differences in packaging between these two classes of chromatin, where euchromatin is more accessible to MNase digestion than heterochromatin [59]. Attempting to exploit this feature, we tried to separate euchromatin and heterochromatin in the S_1_ and S_2_ supernatants, respectively, resulting from a limited MNase digestion (**Figure 4a**) [35]. This MNase preparation also yields a pellet fraction that is thought to be enriched in ‘matrix’-containing chromatin [38], [60]. As shown by SDS-PAGE, S_1_, S_2_ and the pellet fractions were enriched in different chromatin proteins (**Figure 4b**). As expected, euchromatin was mostly digested to mononucleosomes, while heterochromatin was digested into larger DNA oligomers (**Figure 4c**), consistent with a more open euchromatin structure. DNA in the pellet remained in even larger fragments (**Figure 4c**).

**Figure 5:**
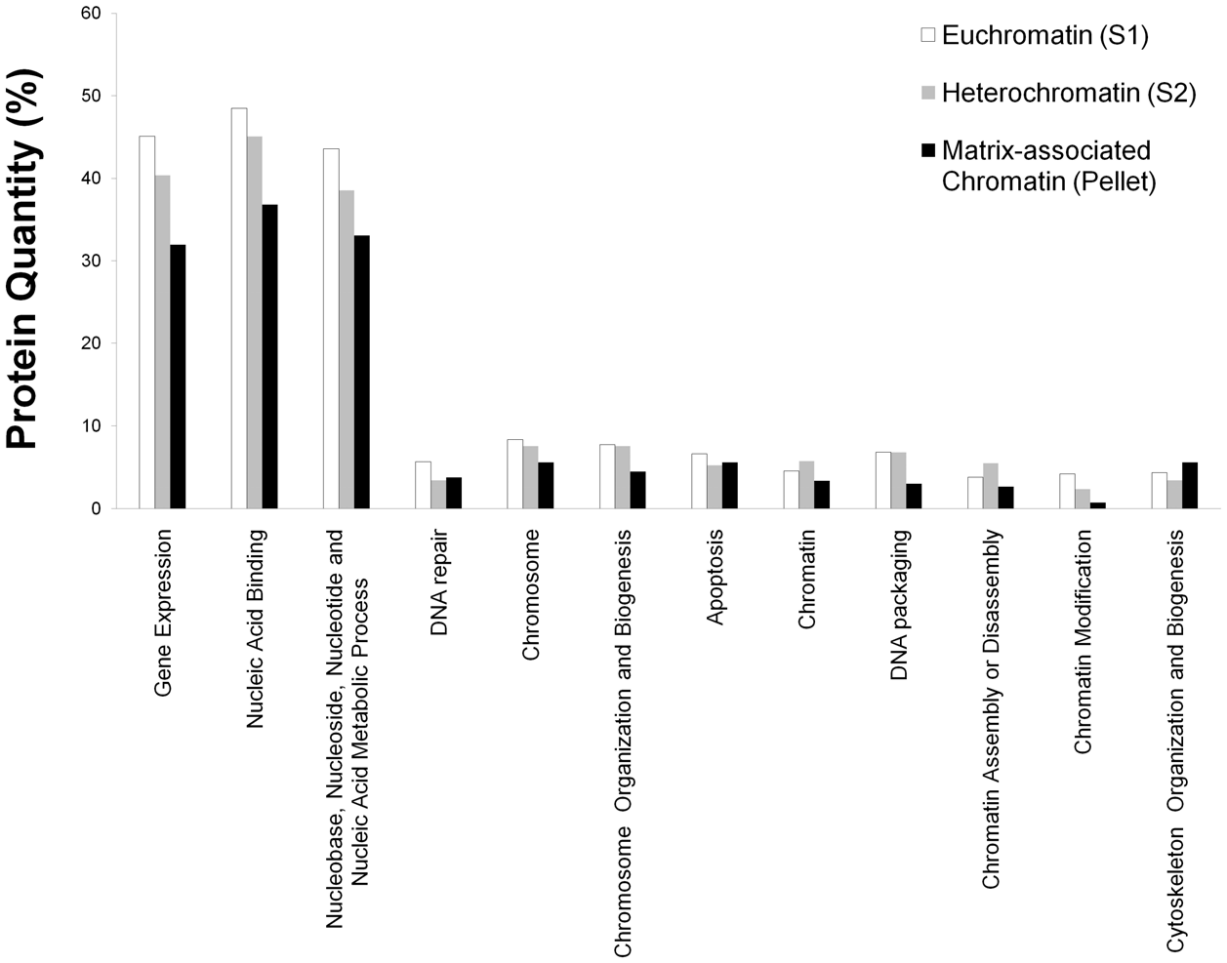
Functional categorization of euchromatin and heterochromatin proteins. The white column corresponds to euchromatin, while the gray and black columns correspond to heterochromatin and matrix-associated chromatin respectively. Protein quantity refers to the percentage of proteins in each category with respect to the total number proteins found in each particular fraction.

To identify the chromatin proteins in the euchromatin or heterochromatin fractions, we analyzed two biological replicates of the S_1_, S_2_ and pellet fractions using MS, reporting only proteins detected in both biological replicates. We found a total of 751 unique proteins between all three fractions, where the majority (691 proteins) were also found in our total chromatin surveys. Out of the 530, 384, and 269 proteins in the euchromatin, heterochromatin, and pellet fractions respectively, 274 and 103 were detected only in euchromatin and heterochromatin respectively, following subtractive analysis. There were 228 proteins shared between euchromatin and heterochromatin (**Figure 4d**). As before, the majority of the proteins identified in all three fractions correspond to nuclear proteins, while less than 15% are categorized as cytosolic, mitochondrial, ribosomal and cytoskeletal (data not shown). We again found that approximately 15% of the proteins found in each fraction could not be annotated. Interestingly, we found that the pellet fractions have a higher percentage of cytosolic and mitochondrial proteins than the S_1_ and S_2_ fractions while it also has a lower amount of non-annotated proteins. As we did for the total chromatin analysis, we checked the functional annotation and categorization of the identified proteins (**Figure 5**). The largest groups of proteins in all fractions are involved in DNA processes, such as gene expression, nucleic acid binding and their metabolic processes. We find the pellet is less enriched in these proteins than euchromatin and heterochromatin fractions.

Among the proteins found enriched in the euchromatin fraction following subtractive analysis, we found TAF 2N isoform 2 whose biological function is consistent with this finding (**Data S4**). In the heterochromatin enriched fraction, we found proteins such as Aurora B kinase, which has a role in marking silent chromatin through phosphorylation of H3S10 [27], [61]. Among the proteins detected in both euchromatin and heterochromatin is the DEK oncogene product (**Data S4**). DEK is an abundant and ubiquitous chromatin protein, which preferentially binds to superhelical and cruciform DNA, and induces positive supercoils into closed circular DNA, and has been suggested to function as an architectural protein in chromatin, akin to HMG proteins [62]. We should note that these preparations are very crude biochemical fractionation methods, and the large overlap of proteins between fractions is presumably due to euchromatin fractions being potentially contaminated with the more abundant heterochromatin portion. Nevertheless, these results seem fairly reproducible through biological replicates. Interestingly, recent results do demonstrate the possibility of overlap of euchromatic and heterochromatic components and chromatin modifications including RNA Polymerase II complex members [49], [63], [64], [65], thus several proteins may be present (but not necessarily active) in both chromatin regions.

### Global histone codes enriched in euchromatin and heterochromatin fractions

**Figure 6:**
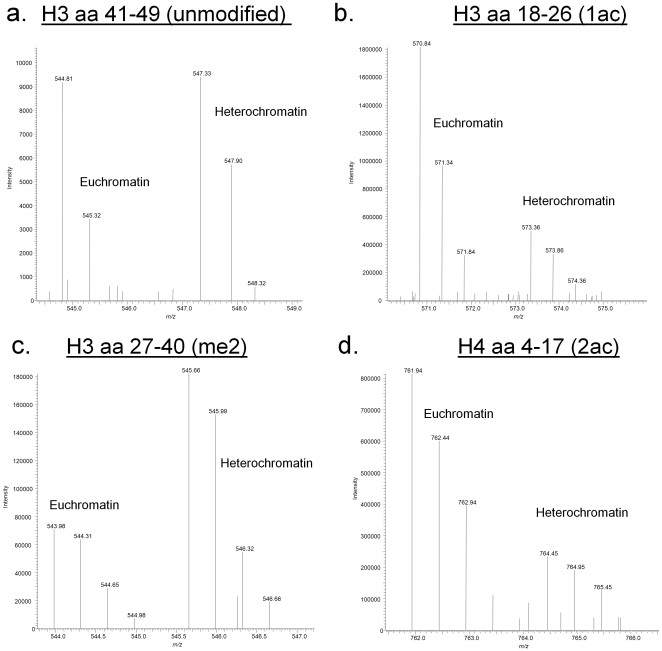
Quantitative comparison of selected histone PTMs in euchromatin (d_0_-labeled) and heterochromatin (d_5_-labeled). (a.) Full mass spectrum for the 2+ charge state of an intrinsically unmodified H3.1 peptide (aa 41–49), (b.) Full mass spectrum for the 2+ charge state of a monoacetylated H3.1 peptide (aa 18–26, H3K18), (c.) Full mass spectrum for the 3+ charge state of the H3.1 dimethylated peptide (aa 27–40, H3K36), (d.) Full mass spectrum for the 2+ charge state of a diacetylated H4 peptide (aa 4–17) Labels specify the fraction represented by each peak. *m/z* values are indicated for each peak.

**Figure 7:**
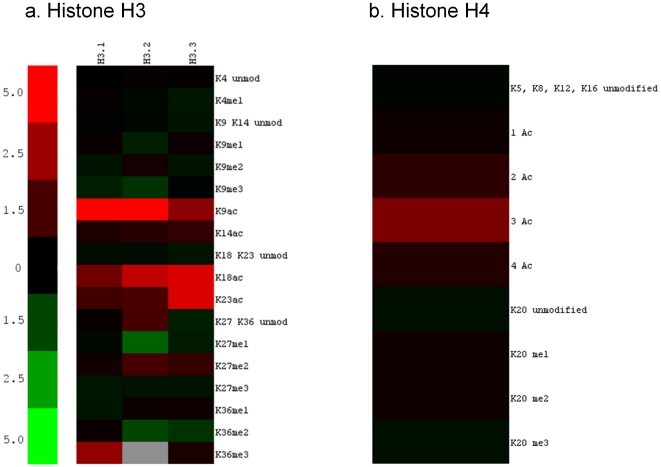
Heat map depicting the ratio of histone H3 and H4 PTMs abundances in euchromatin relative to heterochromatin. Heat maps for histone variants H3.1, H3.2 and H3.3 (a.) and histone H4 (b.). The scale indicates the fold change between a given PTM abundance in euchromatin versus heterochromatin. Red indicates an enrichment of a given PTM in euchromatic regions while green corresponds to enrichment in heterochromatin.

Histones are a major protein component of chromatin, and thus we aimed to validate our crude biochemical separation of euchromatin and heterochromatin by characterizing the abundant PTMs on histone H3 variants and H4 from the euchromatic and heterochromatic enriched fractions using quantitative mass spectrometry (**Figure S2**). We chose to analyze the H3 variants separately because each may be correlated with different transcriptional states, as recently shown by the genomic analysis of the location of mammalian H3 variants [66]. For quantitative investigations of the potential PTM differences amongst the H3 variants, we turned to using a chemical derivatization approach that incorporates stable isotopic labeling onto the histone peptides [43]. Specifically, light (d_0_) and heavy (d_10_) propionic anhydride are used to isotopically label peptides from two samples for relative quantification between the samples. In these experiments, we label peptides from histone H3 extracted from euchromatin with one isotopic label, and histone H3 peptides from heterochromatin with the other isotopic label (two biological replicates). An example of these experiments with peptides from histone H3.1 and H4 is shown in **Figure 6**. The [M+2H]^2+^ ion from the H3 41–49 peptide (YRPGTVALR), which we do not observe to be modified, can be used to gauge equal loading between samples (**Figure 6a**). As expected, histone H3 acetylation levels are enriched in the euchromatin samples, as previously shown from *Drosophila* chromatin [66]. This can be visualized in **Figure 6b** with H3K18 acetylation levels on the 18–26 doubly charged peptide, KQLATKAAR. This particular modification has been described to mainly reside in the region surrounding transcription start sites [67]. Some marks were discovered to be enriched in heterochromatin samples, as is revealed in **Figure 6c** for the [M+3H]^3+^ ion peptide corresponding to H3K36me2 (KSAPATGGVKKPHR). The function of mono- and dimethylation of lysine residues on histones is not well understood compared to the role of lysine trimethylation. Therefore, our data on many of these marks such as the H3K36me2 may help elucidate their role in chromatin. We used this labeling to observe the differential expression of histone marks between euchromatin (i.e. labeled with d_0_) and heterochromatin (i.e. labeled with d_10_), and verified the trends by performing reverse labeling of the two fractions, with results for histone H3 and H4 presented in **Figure 7**.

Our quantitative proteomics analyses found that monoacetylation of H3K9, H3K18 (i.e. H3K18ac1K23un), and H3K23 (i.e. H3K18unK23ac) seems to be increased in euchromatin, while dimethylation of H3 on K36 is increased in heterochromatin not only in for the H3.1 variant (**Figure 6**), but also for all H3 variants (**Figure 7a**). Additionally, H3K36me3 is more abundant in euchromatin, while H3K27me3 is more abundant in heterochromatin (**Figure 7a**). Again, these trends are consistent with current knowledge on the epigenetic function of these PTMs, where H3K36me3 is associated with transcriptional elongation and activation while H3K27me3 is associated with transcriptional silencing [68]. In addition, there does seem to be some variant specific differences in the expression of certain PTMs. For example, trimethylation on H3K9 is decreased in euchromatin for H3.1 and H3.2 compared to H3.3. This K9 modification is widely regarded as a heterochromatic/silencing mark, and thus it is not surprising for it to be decreased on these variants from genomic regions containing potentially more active genes [69]. Similarly, H3K4me1 is slightly increased in heterochromatin enriched H3.3; this is consistent with a report that monomethylation of K4 is a mark for silenced euchromatin [70]. In theory, it could be possible that H3K4me1 would become a mark in heterochromatin as silenced euchromatin becomes part of it. H3K27me1 and H3K9me1 are more enriched on the H3.2 variant extracted from heterochromatin samples. The unmodified K27 and K36 states are enriched on H3.2 from euchromatin, but are decreased slightly in euchromatic H3.3, whereas H3K36me1 levels only seem to decrease on H3.1 in euchromatin. In short, enrichment for most heterochromatic marks appears to be more pronounced for H3.2 than for H3.1 or H3.3. These results support our separation of euchromatin and heterochromatin, while potentially supporting the H3 “barcode model” which postulates that histone H3.1 localizes to constitutive heterochromatin, histone H3.2 to facultative heterochromatin, and H3.3 to euchromatin [71]. However, when we classified these modifications based on their epigenetic function and determined the probability that gene activating- or silencing-associated modifications are more significantly enriched in the euchromatic or heterochromatic preparations, we found that the H3 variant data did not reach statistical significance (*p*>0.05).

Our mass spectrometric analysis of histone H4 from the different genomic samples also found all-acetylated forms of H4 to be enriched in euchromatin, particularly di and tri-acetyl (**Figure 6d** and **Figure 7b**). This result is expected since histone acetylation has been long correlated with transcriptional activation [72]. We also found that mono-acetylation occurs mostly on K16, di-acetylation occurs primarily on K12 and K16 while tri-acetylation occurs on residues K8, K12 and K16 (data not shown). These experiments also revealed monomethylation of K20 to be slightly enriched in euchromatin, whereas trimethylation on K20 to be slightly increased in heterochromatin (**Figure 7b**). When we classify the H4 modifications based on their epigenetic function, namely gene activation or silencing, we found that the gene activating PTMs (i.e. all acetylation states, H4K20me1) and silencing PTMs (i.e. H4K20me2, H4K20me3) were significantly enriched in the S_1_ and S_2_ fractions respectively (*df* = 1, *p* = 0.0476). Altogether, we find some histone PTMs to be enriched in euchromatic and heterochromatic regions and at the same time we also find that there is some overlap in the histone codes of these domains. This suggests that gene activation or silencing may not be constituted by discrete on/off states in terms of histone PTM patterns, and that combinations of histone PTMs may play a larger role in modulating transcriptional states than any single modification alone.

### Conclusion

To define the biological specificity and proteomic extent of three distinct chromatin preparations, we characterized their resulting protein fractions using MS-based proteomics. Our results demonstrate the fact that a decision between biological specificity and broadness of characterization must be made in selecting a chromatin purification method. By way of this analysis, we have also contributed information towards the annotation of the human Chromatome. We identified over 1,900 proteins in the chromatin preparations (counting contaminant proteins), including 193 previously uncharacterized proteins. Our list of results includes highly abundant proteins, such as histones, and lower abundance proteins including histone modifying enzymes and transcription factors. A large proportion of the proteins are known to be involved in DNA templated processes, such as DNA repair and gene regulation. Furthermore, a significant amount of the proteins, including non-histones were also identified as being covalently modified with modifications such as methylation and acetylation. We also crudely separated eu- and heterochromatic protein sub-fractions and corroborated this separation through the parallel quantitative analysis of histone PTMs. In addition to providing new information on the particulars of different chromatin purification methods, we believe this work may pave the way for new discoveries involving the higher-order structure and function of chromatin.

## Supporting Information

Figure S1 Top 10 most abundant proteins identified in the Total Extraction of Chromatin measured through Normalized Spectral Abundance Factors.
(TIF)

Figure S2 Graphic summary of histone H3 and H4 modifications enriched in euchromatin or heterochromatin.
(TIF)

Data S1 Spreadsheet containing all peptides identifying proteins found in all chromatin fractions.
(XLSX)

Data S2 Spreadsheet containing Normalized Spectral Abundance Factors for chromatin proteins.
(XLS)

Data S3 Spreadsheet containing post-translational modifications (PTMs) found on chromatin proteins.
(XLS)

Data S4 Spreadsheet containing Normalized Spectral Abundance Factors for euchromatin and heterochromatin extracted proteins.
(XLS)
