# Supplementary figures and images for: Proteomic Interrogation of Human Chromatin
Source: PLoS One. 2011 Sep 14;6(9):e24747. doi: 10.1371/journal.pone.0024747 (PMC3173473; doi:10.1371/journal.pone.0024747)

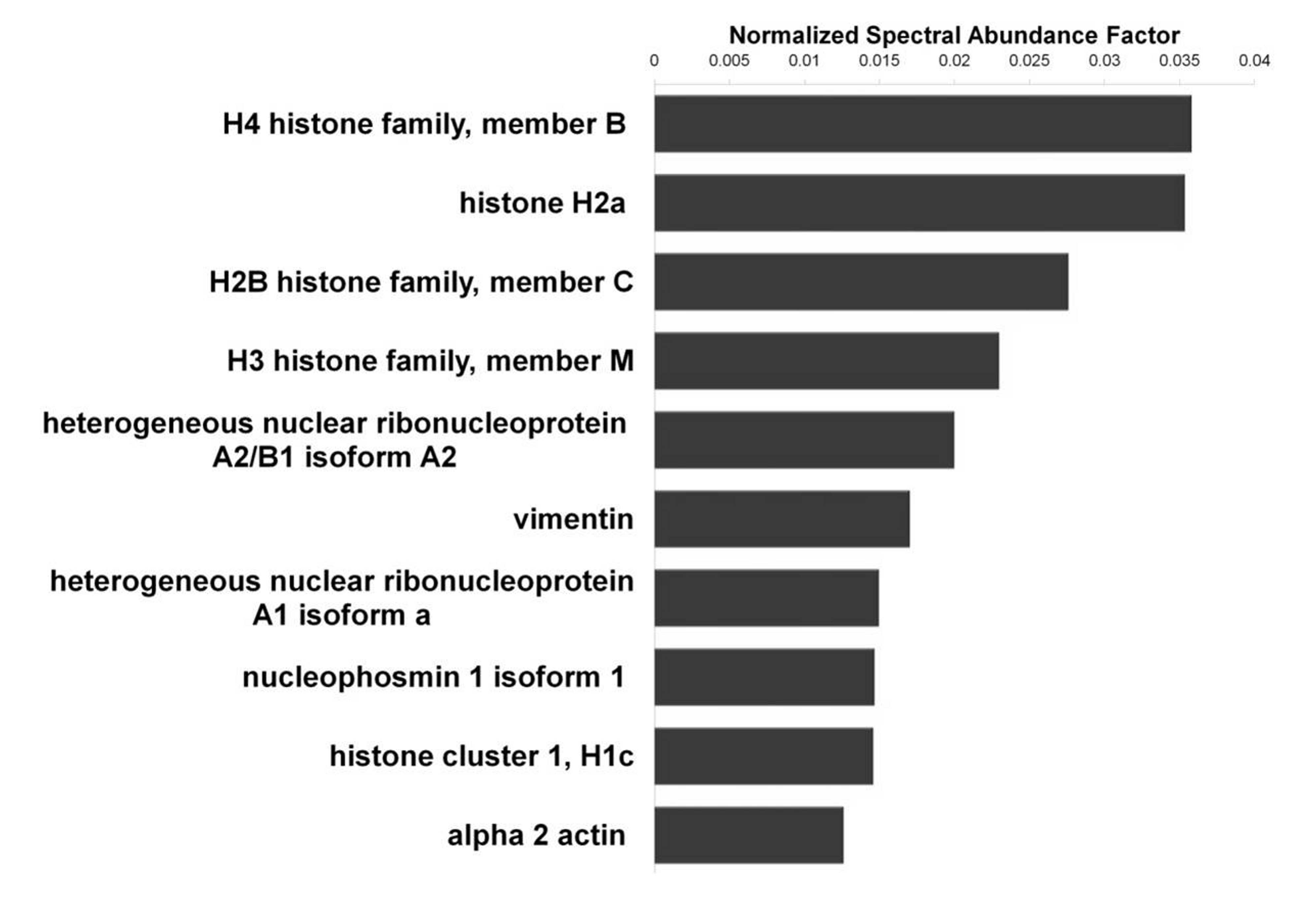

Supplement: Figure S1 — Top 10 most abundant proteins identified in the Total Extraction of Chromatin measured through Normalized Spectral Abundance Factors. (TIF) [file pone.0024747.s001.tif]

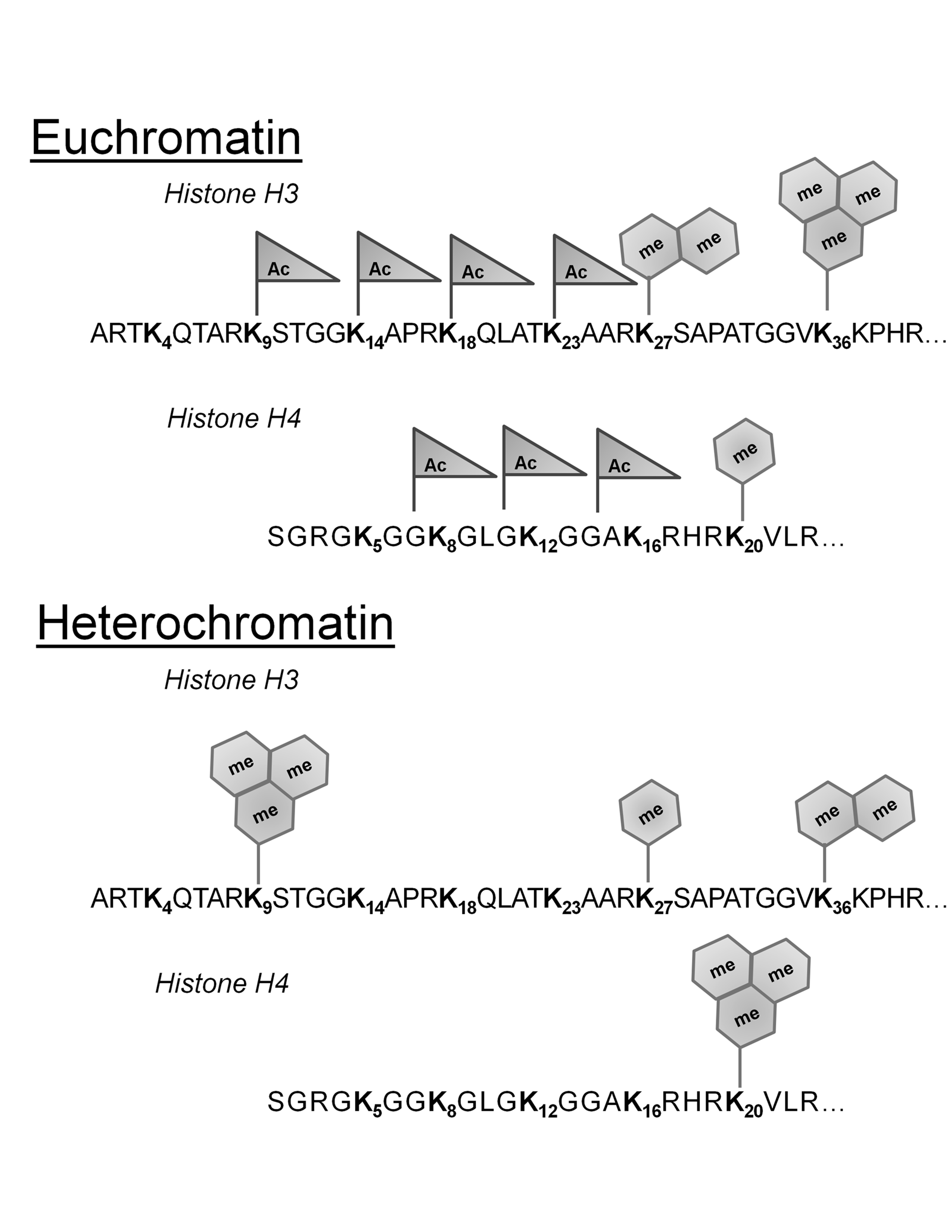

Supplement: Figure S2 — Graphic summary of histone H3 and H4 modifications enriched in euchromatin or heterochromatin. (TIF) [file pone.0024747.s002.tif]
